# Clinical Outcome of Multicentric Lymphoma Treated with Cyclophosphamide, Doxorubicin, Vincristine, and Prednisolone (CHOP) in Small Breed Dogs

**DOI:** 10.3390/ani14202994

**Published:** 2024-10-17

**Authors:** Tae-Hee Kim, Woo-Jin Song, Min-Ok Ryu, Hyun-Tae Kim, Aryung Nam, Hwa-Young Youn

**Affiliations:** 1 Laboratory of Veterinary Internal Medicine, Department of Veterinary Clinical Science, College of Veterinary Medicine, Seoul National University, Seoul 08826, Republic of Korea; bighee92@naver.com (T.-H.K.); apple963@snu.ac.kr (M.-O.R.); 2 Laboratory of Veterinary Internal Medicine and Research Institute of Veterinary Medicine, College of Veterinary Medicine, Jeju National University, Jeju 63243, Republic of Korea; ssong@jejunu.ac.kr; 3 Department of Clinical Sciences and Advanced Medicine, School of Veterinary Medicine, University of Pennsylvania, Philadelphia, PA 19104, USA; petfoodis@gmail.com; 4 Department of Veterinary Internal Medicine, College of Veterinary Medicine, Konkuk University, Seoul 05029, Republic of Korea

**Keywords:** lymphoma, CHOP protocol, small breed dog, survival time, prognostic factors

## Abstract

Lymphoma is one of the most common malignant tumors in dogs. Combination chemotherapy with vincristine, cyclophosphamide, doxorubicin, and prednisolone (CHOP) is the most effective treatment for multicentric lymphoma. Previous studies have evaluated the response of large dogs to CHOP treatment and identified prognostic factors; however, studies on small dogs are lacking. In this study, we investigated the outcomes and prognostic factors for small dogs with multicentric lymphoma treated with CHOP. The responses of patients to CHOP treatment were assessed, and 54.3% were evaluated as being in complete remission (CR), 31.4% in partial remission (PR), and 14.3% in no remission (NR). The overall response rate was 85.7%. The median survival times for CR, PR, and NR patients were 683 days (85–1496 days), 241 days (15–777 days), and 119 days (61–308 days), respectively. Among the CR patients, survival was longer under the following conditions: age under 10 years (*p =* 0.011), no cardiovascular heart disease (*p =* 0.046), and no history of hospitalization due to side effects from chemotherapy (*p =* 0.002). These results might help clinicians build treatment plans for multicentric lymphoma in small breed dogs.

## Simple Summary

This study aimed to evaluate the outcomes and prognostic factors for multicentric lymphoma in 38 small breed dogs (weighing under 15 kg) treated with combination chemotherapy with vincristine, cyclophosphamide, doxorubicin, and prednisolone between January 2014 and May 2020. Significant factors for longer mean survival time in the complete remission group included being under 10 years old, no cardiovascular heart disease, and no prior hospitalization due to chemotherapy side effects. These results will help confirm the clinical characteristics and outcomes in small breed dogs with lymphoma and provide insights into the most effective treatments and prognosis for these animals.

## 1. Introduction

Lymphoma is one of the most common tumors, accounting for 83% of hematopoietic neoplasia in dogs and comprising 7–24% of all tumors [1]. Lymphoma is caused by the abnormal proliferation of malignant lymphoid cells and occurs mainly in lymphoid tissues such as the lymph nodes and spleen [2]. Lymphomas are classified as multicentric, alimentary, and mediastinal; as such, depending on the anatomical location, of these, multicentric lymphoma accounts for 80% of all cases. Although the etiology of lymphoma is not well known, infectious, genetic, environmental, and immunological factors are associated with its prevalence [1,2].

The multi-drug combination of cyclophosphamide (C), doxorubicin (H), vincristine (O), and prednisolone (P) (CHOP) is one of the most recommended chemotherapy treatments for lymphoma [3]. Without treatment, the life expectancy is 4–6 weeks, but most patients administered the CHOP protocol show a positive response and have a survival time of 10–14 months [2,4]. Many studies have examined the therapeutic response and prognostic factors of patients treated with CHOP. Through these studies, stage, substage, immunophenotype, and the presence of hematological abnormalities (anemia or thrombocytopenia) and chronic inflammation have been identified as prognostic factors [4,5,6,7,8,9].

Morphological and physiological differences exist among dog breeds, resulting in differences in metabolic rate and lifespan [10,11,12]. Most previous studies of the CHOP protocol in dogs have focused on large breeds such as boxer, shepherd, and retriever. However, an assessment of treatment response in small breed dogs is lacking. Additionally, few studies have been conducted on changes in survival time due to the presence of other diseases. Underlying conditions can affect the metabolism and secretion of chemotherapy drugs. It is useful, therefore, to evaluate the effects of underlying diseases including cardiovascular heart disease (CVHD), hormonal abnormalities (such as hyperadrenocorticism), and liver failure on prognosis [13].

The aim of this study was to evaluate the outcomes and prognostic factors in multicentric lymphoma patients who were administered the CHOP protocol, focusing on small breed dogs weighing under 15 kg.

## 2. Materials and Methods

### 2.1. Study Population

This retrospective study was conducted using medical data for all patients diagnosed with lymphoma at Seoul National University Veterinary Medical Teaching Hospital (SNU VMTH) between 1 January 2014 and 31 May 2020. Patient information was recorded with the owners’ consent in the electronic charting program (E-friends; Pet Network Veterinarian, Seoul, Republic of Korea).

### 2.2. Inclusion and Exclusion Criteria

Small breed dogs weighing under 15 kg that were diagnosed with multicentric lymphoma based on clinical signs, physical examination, blood analysis, imaging, and fine needle aspiration (FNA) of lymph nodes were included in this study. Of the 74 patients that met these criteria, 31 dogs that did not receive any treatment (*n* = 17) or received chemotherapies other than CHOP-based protocols (*n* = 14) were excluded. Follow-up data were unavailable for 5 dogs. Therefore, 38 dogs were included in the analysis.

### 2.3. Medical Records

Patient information (breed, sex, and birth), medical history, and concurrent disease were collected from the owner and recorded in the e-chart. We examined each patient’s general status, including body weight, vital signs (blood pressure, heart rate, respiratory rate, and temperature), and any abnormal findings from head to tail. All body surface lymph nodes were palpated and if any were noticeably enlarged, we measured them with calipers. Blood analysis, including complete blood count, serum chemistry, and electrolytes, was conducted. Abdominal and thoracic radiographs and abdominal ultrasound were performed to examine intraperitoneal lymph nodes and metastasis. If enlarged intraperitoneal lymph nodes or abnormal nodules were found in the liver or spleen during abdominal ultrasound, additional ultrasound-guided FNA was performed for staging patients.

The stage and substage of patients were determined using the world health organization clinical staging criteria for lymphoma in domestic animals. According to the criteria, infiltration of abnormal lymphoid cells to liver, spleen, or bone marrow was a crucial consideration for determining stage. However, assessment of the liver, spleen, or bone marrow with FNA or biopsy was not performed for all patients. Although ultrasonographic findings, such as “honeycomb signs” in the spleen or liver, could be considered evidence of infiltration, they have a high rate of false negatives and false positives without FNA or biopsy [14]. For these reasons, though patients had abnormal morphologies in their spleen, liver, or both, they were classified as stage 3. Alternatively, abnormalities of peripheral blood such as leukocytosis, lymphocytosis, thrombocytopenia, and findings of moderate to large lymphocytes in the blood smear were enough to determine stage 5, without bone marrow biopsy [15]. Immunophenotype was determined by fluorescence activated cell scanning (FACS) or polymerase chain reaction for antigen receptor rearrangements (PARR). Patients that were diagnosed with lymphoma at a local animal hospital (LAH) and transferred to SNU VMTH were evaluated using the same methods, based on the medical records received from LAH.

### 2.4. CHOP or L-CHOP Protocol

All of the dogs were treated with a modified 25-week University of Wisconsin–Madison (UW-25) chemotherapy protocol based on CHOP, which includes L-asparaginase, cyclophosphamide, doxorubicin, vincristine, and prednisolone (PDS) (*n* = 13) or UW-25 without L-asparaginase (*n* = 25). L-asparaginase (400 U/kg; Leunase injection, Kyowa Kirin, Tokyo, Japan) was administered subcutaneously in the first week of the protocol. For the first four weeks of the induction phase, vincristine (0.5–0.7 mg/m^2^, weeks 1 and 3; vincristine sulfate injection, Hospira Australia Pty Ltd., Mulgrave, Australia), cyclophosphamide (250 mg/m^2^, week 2; Endoxan, Baxter Oncology GmbH, Halle, Germany), and doxorubicin (1 mg/kg, week 4; Adriamycin, Pfizer, Milan, Italy) were administered intravenously. At the same time, PDS (Solondo, Yuhan Corp., Seoul, Republic of Korea) was administered orally, with the dose tapering gradually from 40 mg/m^2^ to 10 mg/m^2^ at weekly intervals. After a week of rest, the treatment was repeated from week six, without oral PDS administration. The maintenance phase then followed. The injection interval was extended by two weeks, and it took 25 weeks to finish the entire protocol. The exact dosage and schedule are shown in Supplementary Table S1.

### 2.5. Evaluation of Response

All patients were classified using the following categories: complete remission (CR), no evidence of disease in any lymph nodes, including normalization of size (under 10 mm); partial remission (PR), the sum of the long diameter of enlarged lymph nodes decreased by more than 30% compared to that before chemotherapy; progressive disease (PD), the sum of the long diameter increased by more than 20% or new lesions appeared; stable disease (SD), did not belong to any of the other three categories (CR, PR, or PD). The presence and size of lymph nodes were assessed through physical examination, radiography, and ultrasonography. We considered SD and PD together as no remission (NR). Survival time was the time from diagnosis to death. Evaluation of the survival time of each patient was based on the e-chart, but if medical records were omitted, we called the client and took new information over the phone.

### 2.6. Statistical Analysis

GraphPad prism (version 6.01) software (GraphPad, Inc., La Jolla, CA, USA) was used for statistical analysis. Differences between two groups were analyzed using Student’s *t*-test and differences between more than two groups were analyzed using one-way analysis of variance (ANOVA), followed by Bonferroni multiple comparison test. The survival outcomes were analyzed using the Kaplan–Meier method, and comparison of survival curves were analyzed using a log-rank (Mantel–Cox) test. The results are presented as mean ± standard deviation (S.D.). Differences with a value of *p* < 0.05 were considered statistically significant.

## 3. Results

### 3.1. Study Animals

The mean age of patients was 9.84 ± 3.00 years (range, 2–15 years) and the mean body weight was 6.42 ± 3.12 kg (range, 2.34–15.0 kg). The percentage of females (intact (*n* = 4, 10.5%) and spayed (*n* = 18, 47.4%); total (*n* = 22, 57.9%)) was slightly higher than that of males (intact (*n* = 3, 7.9%) and castrated (*n* = 13, 34.2%); total (*n* = 16, 42.1%)) (Table 1). Shih-tzu was the most frequently affected breed (*n* = 11, 28.9%), followed by Maltese (*n* = 7, 18.4%) and Cocker spaniel (*n* = 4, 10.5%). Poodle, Miniature pincher, Yorkshire terrier, and mongrel occupied 5.3% each, and each of the eight breeds had only one patient (Table 2).

### 3.2. Clinical Staging and Laboratory Findings

Twenty-one patients (55.3%) were stage 3 (eighteen were substage a and three were substage b), twelve patients (31.6%) were stage 4 (six were substage a and b, respectively), and five patients (13.2%) were stage 5 (two were substage a and three were substage b) (Table 3).

Except for one dog diagnosed at LAH, whose blood analysis results were lost, complete blood counts for 37 patients were assessed using an ADVIA 2120i hematology analyzer (Siemens, Erlangen, Germany), along with manual white blood cell differentials. The mean white blood cell (WBC) count was 15,257.8 ± 9295.5/µL (reference range: 5200–17,000/µL), the mean of the packed cell volume (PCV) was 38.6 ± 7.6% (reference range: 37.1–57.0%), and the mean platelet count was 28.6 ± 16.7 × 10^4^/µL (reference range: 14.3–40.0 × 10^4^/µL) (Table 4). The PCV and platelet count had no statistically significant differences according to stage; however, the WBC counts of stage 5 patients were significantly higher than stage 3 and 4 patients (*p =* 0.002). Specifically, among five patients who were categorized as stage 5, two patients had high proportion of lymphocytes over 70 percent, with lymphocyte/WBC count ratios (/µL) of 21,405/28,540 and 23,162/31,300, respectively. Pre-treatment blood analysis, including serum chemistry and electrolytes, was also conducted, and the mean and S.D. values according to disease stage are presented in Table 5. Among serum chemistry profiles, blood urea nitrogen (BUN) was statistically significantly low in stage 4 patients (*p =* 0.028). There were no statistical differences in serum sodium or potassium levels, according to the stage.

Among the thirty-eight patients, thirty-one had their immunophenotype assessed. Nine patients were assessed by FACS and nineteen by PARR. For three patients assessed at LAH, the method was unknown. Twenty-seven patients (71.1%) were B cell type and four patients (10.5%) were T cell type. Seven patients were not assessed for their immunophenotype.

### 3.3. Correlation between Survival Time and Results of Blood Analysis and Stages at Diagnosis

At diagnosis, the number of patients was assessed for each variable of complete blood count, serum chemistry, and electrolytes categorized as low, normal, or high (Supplementary Table S2). No differences in median survival time were found based on these variables. In addition, there were no significant differences in survival time with stages, substages, or immunophenotypes at diagnosis (Supplementary Figure S1).

### 3.4. Response to CHOP Protocol

**Figure 1:**
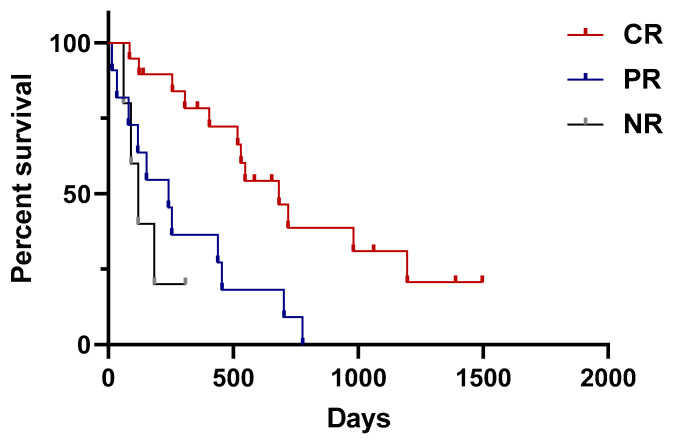
Variation in survival time in each response group. Median survival time is 683 days in CR, 241 days in PR, and 119 days in NR. The CR group survived statistically significantly longer than PR or NR groups (*p =* 0.002, *p =* 0.002 respectively).

Three dogs were excluded from the survival analysis due to death before response evaluation (*n* = 1) and incomplete records of their response to chemotherapy (*n* = 2), leaving 35 dogs for the analysis. Of the patients, 54.3% (19/35) were evaluated as CR; 31.4% (11/35) were PR; 14.3% (5/35) were NR; and a total of 85.7% (30/35) belonged to the CR and PR groups. Median survival time was 683 days (range 85–1496 days) in the CR group, 241 days (range 15–777 days) in the PR group, and 119 days (range 61–308 days) in the NR group (Table 6). The CR group showed a significantly longer survival time than the PR (*p =* 0.002) and NR (*p =* 0.002) groups, but there was no significant difference between the PR and NR groups (*p =* 0.49) (Figure 1).

### 3.5. Factors Affecting Survival Time in CR Patients

**Figure 2:**
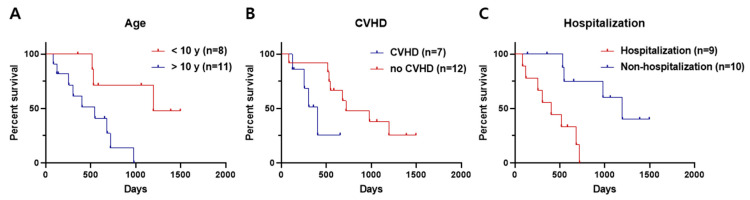
Distribution of different survival times according to influencing factors in the CR group (*n* = 19). (**A**) Ten years of age, (**B**) presence of CVHD, and (**C**) hospitalization made a difference in the survival time. The three factors negatively affected the survival time (*p =* 0.011, *p =* 0.046, and *p* = 0.002, respectively).

In the CR group, dogs under 10 years of age (median 1196 days, range 356–1496 days) had longer survival times than those over 10 years of age (median 547 days, range 85–981 days) (*p =* 0.011) (Figure 2A). The presence of CVHD negatively affected the survival time of patients (non-CVHD group: median 719 days, range 85–1496 days; CVHD group: median 404 days, range 123–654 days) (*p =* 0.046) (Figure 2B). In addition, patients without a history of hospitalization (median 1196 days, range 139–1496 days) had a longer survival time than patients who were hospitalized (median 404 days, range 85–719 days) (*p =* 0.002) (Figure 2C).

## 4. Discussion

Various studies have analyzed the prevalence of lymphoma in dogs, differences in prognosis according to treatment methods, and factors affecting prognosis [5,7,16,17]. Large and small breeds have physiological differences that can result in different responses to disease treatments [11,18,19]. There was no correlation between survival time and stage and substage. This finding suggests that disease progression and the presence of clinical signs at the time of diagnosis are not critical factors for predicting prognosis. One previous study found that stage 4 was a positive prognostic factor [5]; however, in most studies, higher stages of lymphoma or substage b were negative factors [4,7,20,21]. In the present study, assessment of the liver, spleen, or bone marrow through FNA or biopsy for accurate staging was not performed in some dogs, or did not yield meaningful results, which prevented accurate assessment of correlations between the stage and prognosis. Circulating large lymphocytes in the blood, analyzed by CBC equipment and confirmed through manual differential by veterinarians, were considered indicative of stage 5 [15]. Studies have shown that the differentiation between large and small lymphocytes in circulation is an important prognostic factor in canine lymphoma, and the clinical behavior is related to the degree of infiltration of large lymphocytes [22,23]. In addition, there recently has been a report that the stage of canine multicentric lymphoma may shift based on the diagnostic tools used such as physical examination, blood tests, imaging of thoracic X-rays, abdominal ultrasound, and cytological evaluations of the liver, spleen, and bone marrow [24]. Additional studies are required to evaluate the relationship between prognosis and stage in small breed dogs, where accurate staging is performed through cytology or biopsy of the liver, spleen, and bone marrow. B cell phenotypes have been reported as a positive prognostic factor in many previous studies [2,8,17,21]. However, no relationship was identified between survival and immunophenotype assessed by FACS or PARR in this study. It has been reported that the agreement between the two methods was up to 97% for B cell type, and PARR is considered an acceptable assay for determining immunophenotype due to its high specificity [23,24]. Further analysis with a larger number of cases could be beneficial.

The percentages of CR and PR patients were 54.3% and 31.4%, respectively, with a total response rate of 85.7% in this study. Response rates to CHOP-based chemotherapy in dogs with lymphoma have previously been reported to range from 81.3% to 100% [6,17,25,26]. Our results highlight that even small breed dogs show a good response to the treatment of multicentric lymphoma with the CHOP-based protocol. CR patients showed a significantly longer survival time than PR or NR patients. Previous studies involving large breed dogs also have shown that the degree of response to treatment can be a factor affecting survival time [7,8].

We also evaluated factors influencing survival time due to variances within the CR group. Patients under 10 years of age showed longer survival times than those over 10 years. There are fewer precursor cells in the bone marrow of older animals than in young animals, which renders older patients more vulnerable to myelosuppression caused by chemotherapy [27]. Another study found that older dogs responded better to the CHOP protocol, achieving PR rather than CR [6]. As the response to treatment is an important prognostic factor, it tends to decrease with age, which in turn means that the mean survival time may decrease according to age.

Dogs with CVHD had shorter survival times compared to those without CVHD in our study. The CVHD observed in our patients was primarily associated with mitral valve degeneration, which is common in small breed dogs [28]. Among the patients, only one dog was hospitalized due to a heart-related issue, specifically for cardiogenic pulmonary edema. While doxorubicin, a component of the CHOP-based protocol, is known for its potential cumulative cardiotoxicity, with increased serum troponin I levels indicating myocardial damage [29,30], Hallman BE et al. found no significant correlation between mean survival time and clinical cardiotoxicity after doxorubicin treatment [29]. The study predominantly involved large-breed dogs and included various tumor types such as lymphoma, hemangiosarcoma, soft tissue sarcoma, and osteosarcoma. The incidence of clinical cardiotoxicity was notably higher in high-risk breeds predisposed to dilated cardiomyopathy breeds. In older human patients with diffuse large B cell lymphoma and Hodgkin’s lymphoma, pre-existing heart failure was associated with higher lymphoma mortality, with a contributing factor being the reduced use of anthracyclines [31,32]. However, no abnormalities were detected on electrocardiography in our CVHD dogs before or after treatment, and there was no reduction in the doxorubicin dose. Furthermore, we were unable to determine the cause of death for all dogs in the study. Given this, it is difficult to suggest that the shorter survival time in these dogs was influenced by the impact of doxorubicin, and further studies are needed.

Hospitalization due to the side effects of chemotherapy was a negative prognostic factor. Common side effects observed in multidrug chemotherapy regimens for dogs with lymphoma included gastrointestinal GI toxicity, pancreatitis, myelosuppression, and sepsis [33,34,35]. In this study, patients were also hospitalized due to these side effects. Several studies have shown that dose reduction due to myelosuppression after chemotherapy is associated with prolonged survival [4,35]. Side effects can make it difficult to proceed with chemotherapy, necessitating dose reductions or delays in the injection schedule, which might lead to variations in remission and survival time. Consequently, hospitalization due to side effects is considered a negative prognostic factor.

This study has several limitations. First, some variables had to be excluded from the analysis due to missing data. Additionally, assessment of the liver, spleen, or bone marrow with infiltration of lymphoma was not performed for all patients, which may have led to some being classified at a lower stage. Furthermore, immunophenotyping was not conducted for some dogs. While previous studies on the CHOP protocol in canine lymphoma have primarily focused on large-breed dogs, significant survival differences were observed in relation to age, presence of CVHD, and hospitalization in small dogs, despite the relatively low number of patients. Further research involving a larger number of small-breed dogs is needed to analyze the impact of various variables on prognosis of lymphoma.

## 5. Conclusions

In this study, we analyzed the prevalence and prognostic factors of canine multicentric lymphoma in small breed dogs treated with CHOP-based protocol. Response to the chemotherapy protocol acts as an important prognostic factor. Furthermore, age, presence of CVHD, and hospitalization due to side effects from chemotherapy were important prognostic factors within the CR group. These results will be beneficial for confirming the clinical characteristics and outcome information reported in small breed dog lymphoma patients and to help elucidate the best treatment and prognosis for these dogs.

## Figures and Tables

**Table 1:** Characteristics of patients with lymphoma in this study.

Signalments	Value
Age (mean ± S.D. years, range)	9.84 ± 3.00, 2–15
Body weight	6.42 ± 3.12, 2.34–15.0
(mean ± S.D. kg, range)	
Sex	
Intact female (n, %)	4, 10.5
Spayed female (n, %)	18, 47.4
Intact male (n, %)	3, 7.9
Castrated male (n, %)	13, 34.2

**Table 2:** Breeds and numbers of patients with lymphoma in this study.

Breeds	Number of Dogs (%)
Shih-tzu	11 (28.9)
Maltese	7 (18.4)
Cocker spaniel	4 (10.5)
Poodle	2 (5.3)
Yorkshire terrier	2 (5.3)
Miniature pinscher	2 (5.3)
Mongrel	2 (5.3)
Coton de Tulear	1 (2.6)
Dachshund	1 (2.6)
French bulldog	1 (2.6)
Pomeranian	1 (2.6)
Pug	1 (2.6)
Schnauzers	1 (2.6)
Spitz	1 (2.6)
Welsh corgis	1 (2.6)
Total	38 (100)

**Table 3:** Stage and substage distribution of patients in this study.

Variables		Number of Patients (%)
Stage ^(1)^	Substage ^(2)^	
3	a	18 (47.4)
	b	3 (7.9)
4	a	6 (15.8)
	b	6 (15.8)
5	a	2 (5.3)
	b	3 (7.9)

^(1)^ Stage 3: generalized lymph node involvement; stage 4: involvement of liver and/or spleen with stage 1–3; stage 5: involvement of bone marrow with stage 1–4. ^(2)^ Substage a: absence of clinical signs; b: presence of clinical signs.

**Table 4:** Complete blood count analysis of patients in this study.

Variables	Mean ± S.D.
White blood cell ^(1)^ (/µL)	15,257.8 ± 9295.5
Stage 3	13,548.6 ± 8460.6
Stage 4	12,678.2 ± 6514.0
Stage 5	28,112.0 ± 8572.8
Packed cell volume ^(2)^ (%)	38.6 ± 7.6
Stage 3	39.9 ± 6.3
Stage 4	37.3 ± 7.9
Stage 5	35.8 ± 12.3
Platelet ^(3)^ (×10^4^/µL)	28.6 ± 16.7
Stage 3	31.3 ± 17.1
Stage 4	25.5 ± 17.4
Stage 5	24.7 ± 14.8

^(1)^ reference range: 5200–17,000/µL, ^(2)^ reference range: 37.1–57.0%, ^(3)^ reference range: 14.3–40.0 × 10^4^/µL.

**Table 5:** Serum chemistry and electrolyte analysis of patients in this study.

Variables	Stage	Mean	S.D.
(Reference Range)			
ALT ^(1)^	3	60.2	26.2
(5.8–83.3 U/L)	4	113	109.1
	5	111.3	92.6
ALP ^(2)^	3	201.9	209.3
(0–97.9 U/L)	4	1064	1342.7
	5	924	1262.6
BUN ^(3)^	3	18.8	7.8
(9.6–31.4 mg/dL)	4	15.6	7.1
	5	30.6	14
Creatinine	3	0.7	0.2
(0.4–1.3 mg/dL)	4	0.8	0.2
	5	0.8	0.1
Total bilirubin	3	0.1	0.1
(0–0.2 mg/dL)	4	0.2	0.5
	5	0.8	1.5
Total protein	3	6.4	1.2
(5.7–7.5 g/dL)	4	5.9	1.2
	5	5.4	1.4
Albumin	3	3.6	0.7
(2.6–4.4 g/dL)	4	3.3	0.7
	5	2.9	0.6
Sodium	3	146.2	5.9
(145.1–152.6 mmol/L)	4	145.8	2
	5	146.1	2.7
Potassium	3	4.4	0.4
(3.6–5.5 mmol/L)	4	4.5	0.4
	5	4.9	0.2

^(1)^ Alanine aminotransferase, ^(2)^ Alkaline phosphatase, ^(3)^ Blood urea nitrogen.

**Table 6:** Response rate and survival time of each response.

Response	Number of Patients (%)	Median Survival Time (Range)
Complete remission (CR)	19 (54.3)	683 days
		(85–1496 days)
Partial remission (PR)	11 (31.4)	241 days
		(15–777 days)
No remission (NR) (stable disease (SD) and progressive disease (PD))	5 (14.3)	119 days (61–308 days)
Total	35 (100)	306 days (15–1496 days)

## Data Availability

The data that support the findings of this study are available from the corresponding author upon reasonable request.

## Supplementary Materials

The following supporting information can be downloaded at: https://www.mdpi.com/article/10.3390/ani14202994/s1, Supplementary Table S1. Modified 25-week University of Wisconsin–Madison chemotherapy protocol based on CHOP used in this study. Supplementary Table S2. Number of patients which were classified according to blood analysis results. Supplementary Figure S1. Distribution of survival time according to stage (with substage) and immunophenotype at the time of diagnosis. (A) The median survival time was 719, 306, 417.5, 350.5, 382, and 81 days in stage 3a, 3b, 4a, 4b, 5a, and 5b respectively. (B) The median survival time was 455 days in B cell type and 151.5 days in T cell type.
