# Supplementary material for: Clinical Outcome of Multicentric Lymphoma Treated with Cyclophosphamide, Doxorubicin, Vincristine, and Prednisolone (CHOP) in Small Breed Dogs
Source: Animals (Basel). 2024 Oct 17;14(20):2994. doi: 10.3390/ani14202994 (PMC11503739; doi:10.3390/ani14202994)
Supplement: Supplementary file 1 [file animals-14-02994-s001.zip › animals-3176548-supplementary.pdf]

Supplementary Table S1. Modified 25-week University of Wisconsin–Madison chemotherapy protocol based on CHOP used in this study.

**Weeks**	**1**	**2**	**3**	**4**	**6**	**7**	**8**	**9**	**11**	**13**	**15**	**17**	**19**	**21**	**23**	**25**
L-asparaginase (400 U/kg subcutaneous																
Vincristine 0.7 mg/m2 intravenous	○		○		○		○		○		○		○		○	
Cyclophosphamide 250 mg/m2 intravenous or per oral		○				○				○				○		
Doxorubicin 1 mg/kg intravenous				○				○				○				○
Prednisolone (mg/m2)	40	30	20	10												

Supplementary Table S2. Number of patients which were classified blood analysis results.

**Complete blood count**		**Serum chemistry**				**Electrolyte**	
**Variables**	**Number of patients**	**Variables**	**Number of patients**	**Variables**	**Number of patients**	**Variables**	**Number of patients**
WBC	37	ALT	30	Total bilirubin	30	Sodium	33
Leukopenia	1	Low	0	Low	0	Hyponatremia	12
Normal	23	Normal	21	Normal	25	Normal	19
Leukocytosis	13	High	9	High	5	Hypernatremia	2
PCV	37	ALP	30	Total protein	28	Potassium	33
Anemia	17	Low	0	Low	11	Hypokalemia	0
Normal	20	Normal	12	Normal	15	Normal	33
		High	18	High	2	Hyperkalemia	0
Platelet	36	BUN	25	Albumin	30		
Thrombocytopenia	6	Low	3	Low	4		
Normal	23	Normal	19	Normal	25		
Thrombocytosis	7	High	3	High	1		
		Creatinine	25				
		Low	0				
		Normal	25				
		High	0				

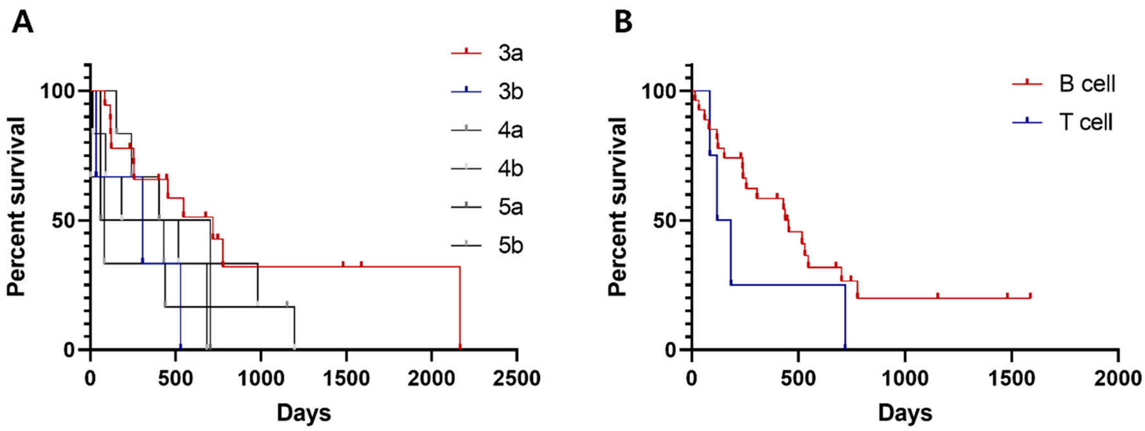

Supplementary Figure S1. Distribution of survival time according to stage (with substage) and immunophenotype at the diagnosed time. (A) The median survival time was 719, 306, 417.5, 350.5, 382, and 81 days in stage 3a, 3b, 4a, 4b, 5a and 5b respectively. (B) The median survival time was 455 days in B cell type and 151.5 days in T cell type.
